# Meibomian gland dysfunction in Sjögren's disease

**DOI:** 10.3389/fmed.2025.1613263

**Published:** 2025-08-07

**Authors:** Esther N. Anuwa-Amarh, Jillian F. Ziemanski

**Affiliations:** Department of Optometry and Vision Science, School of Optometry, University of Alabama at Birmingham, Birmingham, AL, United States

**Keywords:** Sjögren's syndrome, Sjögren's disease, dry eye, MGD, pathophysiology, evaporative dry eye, autoimmune dry eye

## Abstract

For many years, lacrimal gland dysfunction was considered the primary cause of dry eye disease in Sjögren's Disease (SjD). However, recent studies reveal that meibomian gland dysfunction (MGD) is also a significant contributor in dry eye associated with SjD. Recent evidence shows severe meibomian gland damage, reduced tear lipid layer thickness, and abnormal tear evaporation rates, which could exacerbate dry eye symptoms in SjD. These findings challenge the traditional view of SjD dry eye as solely aqueous-deficiency and highlight the role of evaporative dry eye in SjD. While the exact mechanisms linking MGD to SjD remain unclear, researchers propose that inflammation, androgen deficiency, and neurological factors may play key roles. Despite these findings, there is limited research on targeted therapies for SjD-related MGD, which may contribute to why many SjD patients do not experience optimum relief with conventional treatments. This report examines the manifestation of MGD in SjD, explores potential pathophysiological mechanisms, and reviews current management strategies aimed at addressing SjD-related MGD, highlighting the need for further research to improve treatment outcomes.

## 1 Introduction

Meibomian gland dysfunction (MGD) is a common ocular surface condition that affects the meibomian glands (MGs). MGs are modified sebaceous glands located within the tarsal plate of the eyelids. They belong to the category of holocrine glands, an often forgotten subset of exocrine glands (1), similar to the more familiar lacrimal and salivary glands. MGs produce meibum which contributes to the lipid layer of the human tear film (2, 3). The lipid layer plays a vital role in reducing evaporation of the tears, enhancing tear film stability, and maintaining the integrity of the ocular surface (4, 5). In MGD, there is an alteration in the quantity or quality of lipids produced, leading to symptoms of dryness, irritation, redness, and blurred vision, significantly impacting the comfort and quality of life of affected individuals (2, 6). MGD is highly prevalent and is considered the leading cause of evaporative dry eye disease (7). Recently, there has been increasing evidence highlighting the co-occurrence of MGD among Sjögren's Disease (SjD) (8–11), challenging the long-established belief that SjD dry eye disease is exclusively due to aqueous deficiency (11–20).

SjD, formerly known as Sjögren's Syndrome (SS), is a chronic autoimmune disease which primarily affects the exocrine glands, most notably the lacrimal and salivary glands (21, 22). Historically, SjD has been classified into “primary” and “secondary” disease based on the absence or presence, respectively, of other autoimmune conditions such as systemic lupus erythematosus (SLE) and rheumatoid arthritis. However, in 2022, the Sjögren's Foundation recommended migrating away from this nomenclature, noting that there is no significant difference between the phenotype of “primary” and “secondary” SjD (23). SjD is marked by the infiltration of T cells and B cells into the exocrine glands, along with the presence of autoantibodies, including anti-Ro/SSA and anti-La/SSB (24). SjD is well-known for its hallmark symptoms of dry eyes and dry mouth, resulting from lacrimal and salivary gland dysfunction, respectively (25). Inflammation induced by the infiltrating immune cells disrupts the lacrimal and salivary gland epithelia, leading to diminished tear and saliva production (26). Beyond these primary target organs, SjD may also affect other exocrine glands, including the MGs (27). While the role of lacrimal gland dysfunction in SjD dry eye has been extensively studied, the involvement of MGs is gaining increased attention as an important yet underexplored component of the disease. Emerging evidence suggests that MGD is prevalent among SjD patients and may also contribute to the severity of dry eye symptoms (8, 28). Considering these current findings, it is important to understand MGD in SjD. This report discusses the manifestations of MGD among SjD patients, explores possible pathophysiological mechanisms, and reviews current management options.

## 2 Manifestation of MGD in SjD patients

SjD is widely known for its effect on the lacrimal glands, leading to aqueous tear deficiency and, ultimately, dry eye disease. This pathophysiological feature has been extensively studied and has shaped the clinical approach to managing SjD dry eye (29). Recently, attention has shifted toward a more combined disease mechanism, emphasizing the role of the meibomian glands in SjD dry eye. As shown in Table 1, studies have increasingly reported MGD as a frequent occurrence in SjD, underscoring its significance in the pathophysiology of the disease. Several studies have demonstrated evidence of MG structural loss in SjD. In an initial study by Shimazaki et al. (10), the meibomian glands of SjD patients were found to be mostly atrophied, showing clear signs of structural damage to the MGs. When compared to age- and sex-matched aqueous-deficient dry eye patients without SjD, SjD patients exhibited higher rates of meibomian gland dropout (57.9 vs. 18.7%, *p* = 0.017) as measured by meibography. Additionally, the authors assessed meibomian gland obstruction by applying digital pressure to the upper eyelid, and the ease at which meibum was expressed was evaluated as follows: grade 0 = clear meibum is easily expressed; grade 1= cloudy meibum is expressed with mild pressure; grade 2 = cloudy meibum is expressed with more than moderate pressure; and grade 3 = meibum cannot be expressed even with hard pressure. It was observed that the incidence of glandular obstruction (grade 3) was significantly higher in SjD patients compared to the dry eye cohorts (38.9 vs. 11.1%, *p* = 0.028). Similarly, Wang et al. (12) observed greater areas of meibomian gland atrophy in both eyes of SjD patients compared to patients with MGD not associated with SjD. This suggests that the damage to the meibomian glands is more extensive and widespread in SjD, potentially due to the underlying autoimmune destruction present in SjD.

**Table 1:** Summary of research papers investigating MGD in SjD.

**Authors**	**Study design**	**Study population**	**Methods**	**Key findings**
Shimazaki et al. (10)	Non-randomized prospective clinical study	27 SjD patients, 29 non-SjD patients	Meibography, tear evaporation measure	Increased tear evaporation rates, higher incidence of MG dropout, and severe gland obstruction in SjD patients compared to non-SjD patients
Goto et al. (30)	Observational cross-sectional study	21 SjD patients, 12 non-SjD aqueous deficient patients	Tear evaporimetry, lipid layer interferometry (DR-1 camera), meibomian gland expressibility	Higher tear evaporation rates and worse MG expressibility in SjD group compared to non-SjD aqueous deficient patients
Menzies et al. (31)	Observational cross-sectional study	11 SjD patients, 10 healthy controls	Meibography, lipid layer interferometry (tearscope plus), NITBUT	Higher MG dropout scores, reduced lipid layer thickness (LLT), and more rapid tear break-up time in SjD patients
Kang et al. (9)	Observational cross-sectional study	31 SjD patients, 30 non-SjD dry eye patients, 35 healthy controls	Meibography (keratograph 5M), NITBUT	Lower NITBUT, higher meibomian gland dropout and reduced MG expressibility in SjD patients compared to non-SjD and healthy controls
Zang et al. (85)	Prospective case-control study	22 SjD patients, 22 non-SjD aqueous deficient dry eye patients	Meibography, meibomian gland expressibility and secretion quality, LLT, TMH, NIKBUT	More severe MG dropout in upper eyelids; higher corneal staining and lower NIKBUT in SjD group compared to control group
Noh et al. (86)	Retrospective cross-sectional study	108 female SjD patients	Meibography, LLT measurement	Strong correlation between SjD duration and MG atrophy; reduced lipid layer thickness (LLT) with longer SjD duration
Villani et al. (33)	Observational cross-sectional study	20 pSjD (primary SjD), 25 sSjD (secondary SjD), 20 MGD, 25 healthy controls	*In vivo* confocal microscopy (IVCM)	Smaller MG diameters, greater density of periglandular inflammatory cells, and lower secretion reflectivity in pSjD and sSjD patients
Wang et al. (12)	Prospective observational study	49 SjD, 52 MGD patients	Infrared meibography, NITBUT, TMH measurement	Increased lid margin score, MG atrophy, reduced number of expressible glands, and poorer secretion quality in SjD group; MGD severity increased after 3 years of diagnosis in SjD patients
Hwang et al. (20)	Observational cross-sectional study	30 SjD patients, 30 non-SjD dry eye	Meibography, LLT measurement	Higher MG dropout and lower maximum LLT in SjD group
Sullivan et al. (51)	Observational cross-sectional study	11 pSjD (primary SjD), 16 sSjD (secondary SjD), 17 healthy controls, 14 MGD	Lid examination, meibomian gland expressibility, and secretion quality	Increased number of blocked meibomian gland orifices and reduced meibum quality in both pSjD and sSjD patients
Gurlevik et al. (16)	Cross-sectional study	30 SjD, 50 healthy controls	Meibography, TBUT, ocular staining, meibomian gland expressibility and secretion quality	Increased MG loss in SjD group which was found to correlate negatively with TBUT and ocular surface staining.
Chen et al. (11)	Cross-sectional study	34 SjD, 32 healthy controls	Meibography, TBUT, lid examination,	Increased MG loss, increased lid abnormality score and decreased TBUT in SjD group compared to healthy controls.

In addition to MG structural impairment, there have been other reports evidencing functional impairment, such as diminished lipid layer thickness (LLT) and rapid tear evaporation rates among SjD patients (20, 30, 31). Menzies and associates recruited 11 SjD patients and 10 age- and sex-matched normal controls. They measured LLT using a Keeler Tearscope Plus (Keeler, Windsor, UK) and assessed tear film stability by measuring the non-invasive tear break-up time (NITBUT). SjD patients had significantly thinner LLTs and more rapid NITBUTs compared to the normal controls. Also, there was a positive correlation between LLT and NITBUT in SjD (*R* = 0.54, calculated *R*^2^ = 0.291, *p* < 0.05), but not in normal controls. A potential limitation of this study is that it only included normal controls and not patients with non-SjD MGD. Despite this shortcoming, the results from this study shed light on the impairment of MGs in SjD. In another study, Hwang et al. compared the meibography parameters with LLT in 30 SjD dry eye patients and 30 non-SjD dry eye patients using the LipiView II interferometer (Johnson & Johnson Inc., New Brunswick, NJ). Meibomian gland dropout was significantly greater in SjD, and the average LLT was significantly lower, compared to the non-SjD dry eye patients.

Another key characteristic of the manifestation of MGD in SjD is its progressive nature. In a retrospective cross-sectional study, Noh and colleagues analyzed MG images from 108 female SjD patients to explore the correlation between the duration of SjD diagnosis and meibomian gland atrophy. The authors defined the duration of SjD diagnosis to be the interval from the time SjD was confirmed by a rheumatologist to the time meibomian gland imaging was conducted. According to the study, there was a strong positive correlation between SjD duration and MG atrophy (*r* = 0.766, calculated *R*^2^ = 0.587 *p* < 0.001). Even after controlling for age, the association remained significant (*r* = 0.559, calculated *R*^2^ = 0.312, *p* < 0.001). This finding highlights that MG atrophy in SjD is independently linked to disease progression, not just aging. Thus, MGs in SjD patients atrophy over time, with longer disease duration leading to more severe gland dropout. This relationship raises an important pathophysiological question: are the meibomian glands directly attacked by the immune system in SjD, or does their degeneration result from inflammatory spillover of the ocular surface caused by lacrimal gland dysfunction? To date, this question has not been thoroughly investigated, creating a significant gap in our understanding of how MGD develops in SjD. In the following section, three potential mechanisms linking MGD to SjD are explored.

## 3 Proposed pathophysiological mechanisms of MGD in SjD

MGD is a common clinical feature of SjD leading to tear instability and evaporative dry eye. The mechanisms behind MGD in SjD are still not fully understood. However, some researchers propose that MGD associated with SjD might be linked to inflammation, hormonal influences, and neurological mechanisms.

### 3.1 Inflammation

#### 3.1.1 Immune cell infiltration

The infiltration and accumulation of lymphocytes within exocrine glands are hallmarks of SjD. These infiltrating immune cells, mostly consisting of T and B cells, have been shown to induce apoptosis and inflammation in acinar epithelial cells (32). One proposed hypothesis suggests that MG atrophy in SjD may be associated with infiltrating immune cells that have been shown to be present within and around the MGs (33–35). Using *in vivo* confocal microscopy, Villani (33) and associates compared the MGs of SjD patients to those of MGD patients without SjD, as well as to those of normal healthy controls. There was increased inhomogeneity in both the periglandular spaces and the acinar walls compared to not only healthy controls, but also to MGD patients without SjD. This inhomogeneity is consistent with inflammatory cell infiltration within the tarsal plate, suggesting that the MGs, a lesser-known exocrine gland, may also be a target organ in SjD. Of particular interest, in this same study, SjD patients exhibited less acinar dilation and lower secretion reflectivity than MGD patients, two findings that imply that MG obstruction and increased meibum viscosity may not be primary mechanisms of gland dropout in SjD. In primary MGD, in the absence of SjD, ductal obstruction is considered a core mechanism of the disease process (36). In SjD, however, the relative lack of obstructive signs may be a sign that SjD-associated MGD originates external to the glandular ducts and perhaps secondary to inflammatory destruction.

In 1990, Pflugfelder et al. (34) enrolled 19 SjD patients, 18 aqueous-deficient dry eye patients, and 18 seborrheic blepharitis patients. Using conjunctival impression cytology, they discovered infiltrating T lymphocytes within the inferior tarsal epithelium, a cytologic feature specific to SjD (37). Using brush cytology, Hikichi found a significant increase in the incidence of lymphocytes in both the temporal bulbar conjunctiva and inferior tarsal conjunctiva of SjD patients. Furthermore, the incidence of lymphocytes in the tarsal conjunctiva was higher than in the bulbar conjunctiva (3.8 ± 1.2% vs. 13.2 ± 6.2%, *p* = 0.048). Anatomically, the tarsal conjunctiva lies in proximity to the meibomian glands, which are embedded in the tarsal plates of the eyelids. Lymphocytes are key players in autoimmune responses, which are central to the pathogenesis of SjD (24, 38–41). It remains to be investigated whether lymphocytic infiltration into the tarsal conjunctiva may cause immune-mediated damage to the MGs, similar to the lacrimal glands.

#### 3.1.2 Local complement dysregulation

Another plausible mechanism that could be involved in inflammatory changes in the MG of SjD patients is the complement system. The complement system is made up of over 30 proteins and is activated via three pathways: the classical, lectin, and alternative pathways (42). Initiated by activated antibody-antigen complexes, the classical pathway is, therefore, the most likely pathway to be altered in autoimmune diseases, such as SjD (43–46). Studies have shown that complement C3 is upregulated in the tears of SjD patients with dry eye (47), indicating local dysregulation of the complement system. Additionally, increased levels of anti-Ro/SSA and anti-La/SSB autoantibodies have been detected in the tears of SjD patients, with their presence correlating with the severity of dry eye symptoms (48). We hypothesize that these autoantibodies could form immune complexes which could activate the classical complement pathway at the ocular surface. Complement activation leads to the generation of pro-inflammatory molecules such as C3a and C5a, which promote chronic inflammation. C5a is a powerful chemoattractant which is involved in the recruitment of immune cells such as neutrophils, monocytes, eosinophils and T-cells to target sites (49). Although the tarsal plate is a rigid tissue, there is a possibility that inflammatory molecules such as matrix metalloproteinases (MMPs), which are upregulated in SjD dry eye (50) could degrade extracellular matrix components of the tarsus, potentially making it easier for recruited cells to infiltrate toward the MGs. The combination of immune cell infiltration, autoantibody production, and complement-mediated inflammation creates a vicious cycle that could exacerbate glandular dysfunction and MG atrophy in SjD. However, while this hypothesis provides a plausible mechanism for meibomian gland damage in SjD, further research is needed to validate these speculations and is actively ongoing.

### 3.2 Hormonal influences

It has been postulated that androgen deficiency could potentially contribute to MGD among SjD patients (51). Reports have shown that women with SjD have significantly lower serum concentrations of androgen precursors, hormones, and metabolites compared to healthy individuals (52, 53). Androgens play a vital role in regulating the structure, function, and lipid production of the MGs (54–57). These hormones exert their effects by binding to androgen receptors located in the acinar epithelial cells of the glands, where they regulate gene expression related to lipid metabolism and activate enzymes essential for lipid synthesis (58–60). Additionally, androgens such as testosterone help regulate genes involved in the keratinization process of the MG (61, 62), helping maintain normal glandular function. This may explain why topical 0.03% testosterone has shown efficacy in treating MGD in clinical trials (63). Also, androgens have been shown to reduce lymphocytic infiltration in the major target organs in SjD (64–66), indicating a possible anti-inflammatory role. Based on these findings, we hypothesize that androgen deficiency could pose a dual risk to MG structure and function. Firstly, the lack of androgens may impair the regulation of MG function by impairing lipid biosynthesis and enhancing keratinization. Secondly, there might be reduced suppression of lymphocytic infiltration into the areas surrounding the MGs. Enhanced activity of immune cells could lead to increased inflammation with subsequent damage to the glands.

It is also worth noting that perimenopausal women, who are at an increased risk of developing SjD and MGD (67), often exhibit reduced androgen levels (68, 69), indicating a possible shared pathway which links MGD to SjD. What remains unclear is whether androgen deficiency in SjD directly influences the severity of MGD. Additional research is needed to clarify the precise role of androgens in the pathogenesis of MGD in SjD patients, as this could provide valuable insights for targeted therapeutic interventions.

### 3.3 Neurological mechanisms

Wang and associates proposed a speculative yet intriguing hypothesis citing that certain neurological or constitutional symptoms of SjD such as depression, insomnia, and fatigue may also lead to MGD due to a reduced blink rate (12, 70, 71). The authors hypothesized that SjD patients experiencing depression, fatigue, or insomnia may exhibit decreased blink rates, potentially due to reduced alertness or a lack of conscious effort to blink. During each blink action, the Riolan muscle together with the orbicularis muscle contract to assist in the delivery of meibum from the ducts unto the lid margin. The reduction in blinking could lead to inadequate meibum secretion which could lead to stasis of meibum and eventually MG obstruction. While this proposed mechanism provides a plausible explanation for how SjD symptoms might indirectly contribute to MGD, it remains a theoretical construct. Further research is needed to confirm whether blinking is indeed a significant contributor to MGD in SjD patients. These speculations, while scientifically grounded, underscore the complexity of the pathophysiology of MGD associated with SjD.

## 4 Management strategies of MGD in SjD

It is well-documented that MGD plays a significant role in the severe dry eye symptoms experienced by patients with SjD (28). This may partly explain why conventional treatments often fail to provide relief for all SjD patients with dry eye. While current research identifies MGD as a major factor in SjD-related dry eye, there is limited information on targeted treatments in this patient population. In this section, we will explore some remedies supported by studies that have been shown to effectively improve MGD in SjD patients, prioritizing those that have been evaluated as first-line or standalone treatments.

### 4.1 Vectored thermal pulsation (VTP)

Vectored thermal pulsation is a procedure which utilizes a combination of heat and pressure to evacuate the contents of blocked MGs with the intent of removing glandular obstruction (72, 73). Studies have shown that VTP may show some potential in alleviating MGD signs and symptoms in SjD patients (74, 75). In a retrospective study, Epitropoulos et al. evaluated the effectiveness of a vectored thermal pulsation treatment, in improving MGD in 59 patients (102 eyes) with suspected SjD, of whom 23 tested positive and 36 tested negative using the Sjö test kit. The Sjö test is a commercially available blood test kit designed to detect novel biomarkers such as salivary gland protein-1, parotid secretory protein and carbonic anhydrase 6 in addition to the traditional biomarkers of SjD: SSA/Ro, SSB/La, RF, and ANA (76, 77). After 8 weeks post-treatment, MGD patients with or without SjD experienced significant improvement in meibomian gland function as measured by meibomian gland secretion (MGS) and dry eye symptoms (OSDI). Similarly, Godin and associates, in a prospective study, treated 14 SjD patients with VTP and followed them up to a year. After 1-year post-treatment, there was significant improvement in MG oil flow score, corneal staining score and conjunctival staining score. Altogether, these studies support that VTP treatment can be successful in treating MGD and dry eye symptoms in SjD patients. In future studies, it will be useful if VTP is investigated in SjD on a larger scale in a randomized clinical trial, considering the sample sizes in these studies were generally small.

### 4.2 Intense pulsed light (IPL)

Intense Pulsed Light has emerged as a promising therapy for non-SjD MGD patients and has been recently proposed as a useful adjunct therapy for SjD dry eye patients (78–81). IPL is a non-laser light therapy which uses broad spectrum light to target inflammation and has been shown to improve MG function among MGD patients. Huo et al. evaluated the effectiveness of IPL combined with meibomian gland expression (MGX) in treating SjD dry eye. In this randomized controlled trial, 50 SjD patients were randomized into two groups (26 in the IPL-MGX group and 24 in the control group—sodium hyaluronate drops only). Significant improvements were observed in the IPL-MGX group, including reduced ocular surface disease index (OSDI) scores, increased NITBUT, and decreased corneal fluorescein staining, with benefits sustained over 15 weeks. Notably, the IPL-MGX group showed better outcomes in MG-related parameters, such as improved meibum quality and reduced eyelid margin abnormalities. In another randomized controlled trial, when IPL was used in conjunction with 0.05% cyclosporine A eye drops, SjD patients showed significant improvements in MG expressibility, MG quality, and lid margin abnormalities compared to the control group (79). Although the mechanism of action of IPL in alleviating MGD symptoms still remains unclear, it has demonstrated promise as an effective therapy for the management of MGD associated with SjD. It is also important to consider that IPL therapy is generally contraindicated in patients with SLE due to photosensitivity (82), especially since SLE often co-presents with SjD. SLE patients often exhibit heightened sensitivity of light, and exposure to broad-spectrum light sources like IPL may trigger cutaneous flares or exacerbate systemic symptoms (82, 83). Therefore, clinicians should carefully consider the potential for additional autoimmune conditions, such as SLE, before initiating IPL for the management of MGD in SjD.

### 4.3 Punctal plugs

Punctal plugs have been widely used as a remedy for aqueous-deficient dry eye by preventing the drainage of aqueous tears down the nasolacrimal duct (84). Most recently, a prospective study by Liu et al. (15) reported that MG function seemed to improve in SjD patients post-treatment, an interesting observation considering that punctal plugs are not a direct treatment of MGD. It was speculated by the authors that inflammation could play a role. They believed that increased inflammation at the ocular surface could cause hyperkeratinization of the ductal epithelium which eventually could enhance gland atrophy, subsequently altering the function of the MGs. It was suggested that punctal plugs may offer adjunctive benefits to MG function by decreasing tear drainage, which might reduce inflammation on the ocular surface and allow recovery of the MGs. This effect is speculative and requires further research.

## 5 Limitations of existing evidence

While current literature supports the presence of MGD in SjD, there are some limitations that should be acknowledged. Many cited studies relied on relatively small sample sizes which could reduce the statistical power and increase the risk of sampling bias (10, 11, 20, 30, 31, 51, 85). This limitation is of great importance especially in a heterogeneous condition like SjD, where disease presentation, severity, and systemic involvement can vary widely. Furthermore, the majority of the studies were observational and cross-sectional in nature (9–12, 16, 20, 30, 31, 33, 51, 85, 86). While they effectively demonstrated that SjD is associated with increased meibomian gland dropout, thinner lipid layer thickness, and more unstable tear films, the underlying mechanisms behind such features could not be assessed due to the study design. Another common shortcoming is the lack of an appropriate “disease control” group, specifically, those who have MGD but do not have SjD (9–11, 16, 20, 30, 31, 85). Without an appropriate disease control group, it is difficult to determine whether the observed MG features are specific to SjD or a reflection of the generic MGD pathology. These methodological constraints highlight the need for larger, prospective, controlled studies to better understand the relationship between MGD and SjD and to guide development of targeted management strategies.

## 6 Conclusion

The traditional belief that lacrimal gland dysfunction is the primary driver of dry eye disease in SjD has been challenged by recent literature. Instead, MGD has been identified as a frequent occurrence among SjD patients and is reported to contribute to severe dry eye symptoms. Despite the high prevalence of MGD in SjD, surprisingly little research has been conducted to explore its underlying pathophysiology. While some hypotheses have been proposed—such as the roles of inflammation, hormonal imbalances, and neurological mechanisms—these speculations are still in the early stages of being investigated and/or validated. As a result, the mechanism behind MGD in SjD remains poorly understood, creating a significant gap in the development of targeted therapies to alleviate MGD-related signs and symptoms in this patient population.

Emerging research suggests potential promise in treatments such as VTP, IPL, and punctal plugs. However, the specific mechanisms by which these therapies might improve MGD in SjD remain unclear. Given these uncertainties, there is an urgent need for research focused on uncovering the underlying cause of MGD in SjD. We propose that future studies should prioritize investigating specific immune pathways that may contribute to MGD pathology, particularly since SjD is an autoimmune condition. Additionally, larger-scale clinical trials are needed to compare the effectiveness of existing MGD therapies in SjD, including VTP and IPL but also other off-label dry eye medications, warm compresses, lid hygiene, doxycycline, etc. By addressing these research gaps, we can move closer to developing more effective treatments to address an often-overlooked aspect of SjD dry eye.

## References

[1] Henriquez AS Korb DR. Meibomian glands and contact lens wear. Br J Ophthalmol. (1981) 65:108–11. 10.1136/bjo.65.2.108 7459311 PMC1039437

[2] Nichols KK Foulks GN Bron AJ Glasgow BJ Dogru M Tsubota K. The international workshop on meibomian gland dysfunction: executive summary. Invest Ophthalmol Vis Sci. (2011) 52:1922–9. 10.1167/iovs.10-6997a 21450913 PMC3072157

[3] Gutgesell VJ Stern GA Hood CI. Histopathology of meibomian gland dysfunction. Am J Ophthalmol. (1982) 94:383–7. 10.1016/0002-9394(82)90365-8 7124880

[4] Nelson JD Shimazaki J Benitez-del-Castillo JM Craig JP McCulley JP Den S. The international workshop on meibomian gland dysfunction: report of the definition and classification subcommittee. Invest Ophthalmol Vis Sci. (2011) 52:1930–7. 10.1167/iovs.10-6997b 21450914 PMC3072158

[5] Holly F Lemp M. Tear physiology and dry eyes. Surv Ophthalmol. (1977) 22:69–87. 10.1016/0039-6257(77)90087-X 335548

[6] Asiedu K Dzasimatu S Kyei S. Impact of meibomian gland dysfunction on quality of life and mental health in a clinical sample in Ghana: a cross-sectional study. BMJ Open. (2022) 12:e061758. 10.1136/bmjopen-2022-061758 36180116 PMC9528660

[7] Schaumberg DA Nichols JJ Papas EB Tong L Uchino M Nichols KK. The international workshop on meibomian gland dysfunction: report of the subcommittee on the epidemiology of, and associated risk factors for, MGD. Invest Ophthalmol Vis Sci. (2011) 52:1994–2005. 10.1167/iovs.10-6997e 21450917 PMC3072161

[8] Srinivasan S Caffery B Harthan JS Acs M Barnett M Johnson-Tong L. Prevalence of meibomian gland dysfunction in Sjogren's syndrome patients. Invest Ophthalmol Vis Sci. (2018) 59:921.29450539

[9] Kang YS Lee HS Li Y Choi W Yoon KC. Manifestation of meibomian gland dysfunction in patients with Sjogren's syndrome, non-Sjogren's dry eye, and non-dry eye controls. Int Ophthalmol. (2018) 38:1161–7. 10.1007/s10792-017-0577-4 28567496

[10] Shimazaki J Goto E Ono M Shimmura S Tsubota K. Meibomian gland dysfunction in patients with Sjogren syndrome. Ophthalmology. (1998) 105:1485–8. 10.1016/S0161-6420(98)98033-2 9709762

[11] Chen X Utheim OA Xiao J Adil MY Stojanovic A Tashbayev B. Meibomian gland features in a Norwegian cohort of patients with primary Sjogren s syndrome. PLoS ONE. (2017) 12:e0184284. 10.1371/journal.pone.0184284 28886085 PMC5590907

[12] Wang Y Qin Q Liu B Fu Y Lin L Huang X. Clinical analysis: aqueous-deficient and meibomian gland dysfunction in patients with primary Sjogren's syndrome. Front Med. (2019) 6:291. 10.3389/fmed.2019.00291 31921869 PMC6914862

[13] Roszkowska AM Oliverio GW Aragona E Inferrera L Severo AA Alessandrello F. Ophthalmologic manifestations of primary Sjögren's syndrome. Genes. (2021) 12:365. 10.3390/genes12030365 33806489 PMC7998625

[14] Danjo Y Hamano T. Observation of precorneal tear film in patients with Sjögren's syndrome. Acta Ophthalmol Scand. (1995) 73:501–5. 10.1111/j.1600-0420.1995.tb00324.x 9019372

[15] Liu T Liu S Gan M He Y Fu H Xu M. Changes of dry eye parameters especially meibomian gland functions after punctal plugs insertion in aqueous-deficient dry eye patients. Front Med. (2022) 9:849700. 10.3389/fmed.2022.849700 35308530 PMC8925321

[16] Gurlevik U Karakoyun A Yasar E. Does Sjogren's syndrome affect only the lacrimal gland in the eye? Time to replace the missing stones. Indian J Ophthalmol. (2021) 69:53–7. 10.4103/ijo.IJO_2383_19 33323573 PMC7926172

[17] Zhao J Manthorpe R Wollmer P. Surface activity of tear fluid in patients with primary Sjögren's syndrome. Clin Physiol Funct Imaging. (2002) 22:24–7. 10.1046/j.1475-097X.2002.00389.x 12003095

[18] Zhao S Le Q. Analysis of the first tear film break-up point in Sjogren's syndrome and non-Sjogren's syndrome dry eye patients. BMC Ophthalmol. (2022) 22:1. 10.1186/s12886-021-02233-6 34980014 PMC8722312

[19] Akpek EK Bunya VY Saldanha IJ. Sjogren's syndrome: more than just dry eye. Cornea. (2019) 38:658–61. 10.1097/ICO.0000000000001865 30681523 PMC6482458

[20] Hwang JM Lee JM Chung SH. Comparison of meibomian gland imaging findings and lipid layer thickness between primary Sjogren syndrome and non-Sjogren syndrome dry eyes. Ocul Immunol Inflamm. (2020) 28:182–7. 10.1080/09273948.2018.1562557 30794472

[21] Kassan SS Moutsopoulos HM. Clinical manifestations and early diagnosis of Sjögren syndrome. Arch Intern Med. (2004) 164:1275–84. 10.1001/archinte.164.12.1275 15226160

[22] Baer AN Hammitt KM. Sjögren's disease, not syndrome. Arthritis Rheumatol. (2021) 73:1347–8. 10.1002/art.41676 33559389

[23] Kollert F Fisher BA. Why Language Matters: Sjögren's v. “Primary/Secondary Sjögren's”: Sjögren's Foundation. (2022). Available online at: https://sjogrens.org/blog/2022/why-language-matters-sjogrens-v-primarysecondary-sjogrens (Accessed April 15, 2025).

[24] Nguyen C Cornelius J Singson E Killedar S Cha S Peck AB. Role of complement and B lymphocytes in Sjogren's syndrome-like autoimmune exocrinopathy of NODB10-H2b mice. Mol Immunol. (2006) 43:1332–9. 10.1016/j.molimm.2005.09.003 16221495

[25] Harley JB. Autoantibodies in Sjögren's syndrome. Sjögren's Syndrome: Clinical and Immunological Aspects. Berlin, Heidelberg: Springer (1987). p. 218–34. 10.1007/978-3-642-50118-0_21

[26] Stern ME Gao J Siemasko KF Beuerman RW Pflugfelder SC. The role of the lacrimal functional unit in the pathophysiology of dry eye. Exp Eye Res. (2004) 78:409–16. 10.1016/j.exer.2003.09.003 15106920

[27] Roguedas A Misery L Sassolas B Le Masson G Pennec Y Youinou P. Cutaneous manifestations of primary Sjogren's syndrome are underestimated. Clin Exp Rheumatol. (2004) 22:632–6. 15485020

[28] Zi C Huang Q Ren Y Yao H He T Gao Y. Meibomian gland dysfunction and primary Sjogren's syndrome dry eye: a protocol for systematic review and meta-analysis. BMJ Open. (2021) 11:e048336. 10.1136/bmjopen-2020-048336 35044322 PMC8719179

[29] Foulks GN Forstot SL Donshik PC Forstot JZ Goldstein MH Lemp MA. Clinical guidelines for management of dry eye associated with Sjögren disease. Ocul Surf. (2015) 13:118–32. 10.1016/j.jtos.2014.12.001 25881996

[30] Goto E Matsumoto Y Kamoi M Endo K Ishida R Dogru M. Tear evaporation rates in Sjogren syndrome and non-Sjogren dry eye patients. Am J Ophthalmol. (2007) 144:81–5. 10.1016/j.ajo.2007.03.055 17509507

[31] Menzies KL Srinivasan S Prokopich CL Jones L. Infrared imaging of meibomian glands and evaluation of the lipid layer in Sjogren's syndrome patients and nondry eye controls. Invest Ophthalmol Vis Sci. (2015) 56:836–41. 10.1167/iovs.14-13864 25574045

[32] Fujihara T Fujita H Tsubota K Saito K Tsuzaka K Abe T. Preferential localization of CD8+ αEβ7+ T cells around acinar epithelial cells with apoptosis in patients with Sjogren's syndrome. J Immunol. (1999) 163:2226–35. 10.4049/jimmunol.163.4.2226 10438965

[33] Villani E Beretta S De Capitani M Galimberti D Viola F Ratiglia R. *In vivo* confocal microscopy of meibomian glands in Sjogren's syndrome. Invest Ophthalmol Vis Sci. (2011) 52:933–9. 10.1167/iovs.10-5995 21169534

[34] Pflugfelder SC Huang AJ Feuer W Chuchovski PT Pereira IC Tseng SC. Conjunctival cytologic features of primary Sjogren's syndrome. Ophthalmology. (1990) 97:985–91. 10.1016/S0161-6420(90)32478-8 1698273

[35] Hikichi T Yoshida A Tsubota K. Lymphocytic infiltration of the conjunctiva and the salivary gland in Sjögren's syndrome. Arch Ophthalmol. (1993) 111:21–2. 10.1001/archopht.1993.01090010023009 8424715

[36] Knop E Knop N Millar T Obata H Sullivan DA. The international workshop on meibomian gland dysfunction: report of the subcommittee on anatomy, physiology, and pathophysiology of the meibomian gland. Invest Ophthalmol Vis Sci. (2011) 52:1938–78. 10.1167/iovs.10-6997c 21450915 PMC3072159

[37] Xu KP Katagiri S Takeuchi T Tsubota K. Biopsy of labial salivary glands and lacrimal glands in the diagnosis of Sjögren's syndrome. J Rheumatol. (1996) 23:76–82. 8838512

[38] Katsifis GE Moutsopoulos NM Wahl SM. T lymphocytes in Sjögren's syndrome: contributors to and regulators of pathophysiology. Clin Rev Allergy Immunol. (2007) 32:252–64. 10.1007/s12016-007-8011-8 17992592

[39] Viau M Zouali M. B-lymphocytes, innate immunity, and autoimmunity. Clin Immunol. (2005) 114:17–26. 10.1016/j.clim.2004.08.019 15596405

[40] Youinou P Saraux A Pers J-O. B-lymphocytes govern the pathogenesis of Sjogren's syndrome. Curr Pharm Biotechnol. (2012) 13:2071–7. 10.2174/138920112802273100 22208653

[41] Zhou H Yang J Tian J Wang S. CD8+ T lymphocytes: crucial players in Sjögren's syndrome. Front Immunol. (2021) 11:602823. 10.3389/fimmu.2020.602823 33584670 PMC7876316

[42] Merle NS Church SE Fremeaux-Bacchi V Roumenina LT. Complement system part I–molecular mechanisms of activation and regulation. Front Immunol. (2015) 6:134383. 10.3389/fimmu.2015.00262 26082779 PMC4451739

[43] Alexander EL Provost TT Sanders ME Frank MM Joiner KA. Serum complement activation in central nervous system disease in Sjögren's syndrome. Am J Med. (1988) 85:513–8. 10.1016/S0002-9343(88)80087-1 3177398

[44] Chen M Daha MR Kallenberg CG. The complement system in systemic autoimmune disease. J Autoimmun. (2010) 34:J276–86. 10.1016/j.jaut.2009.11.014 20005073

[45] Li M Qi Y Wang G Bu S Chen M Yu J. Proteomic profiling of saliva reveals association of complement system with primary Sjogren's syndrome. Immun Inflamm Dis. (2021) 9:1724–39. 10.1002/iid3.529 34516718 PMC8589410

[46] Lin W Xin Z Wang J Ren X Liu Y Yang L. Hypocomplementemia in primary Sjogren's syndrome: association with serological, clinical features, and outcome. Clin Rheumatol. (2022) 41:2091–102. 10.1007/s10067-022-06135-w 35348930 PMC9187545

[47] Li B Sheng M Li J Yan G Lin A Li M. Tear proteomic analysis of Sjögren syndrome patients with dry eye syndrome by two-dimensional-nano-liquid chromatography coupled with tandem mass spectrometry. Sci Rep. (2014) 4:1–6. 10.1038/srep05772 25159733 PMC4145314

[48] Toker E Yavuz S Direskeneli H. Anti-Ro/SSA and anti-La/SSB autoantibodies in the tear fluid of patients with Sjögren's syndrome. Br J Ophthalmol. (2004) 88:384–7. 10.1136/bjo.2003.028340 14977774 PMC1772044

[49] Guo R-F Ward PA. Role of C5a in inflammatory responses. Annu Rev Immunol. (2005) 23:821–52. 10.1146/annurev.immunol.23.021704.115835 15771587

[50] López-Miguel A Tesón M Martín-Montañez V Enríquez-de-Salamanca A Stern ME González-García MJ. Clinical and molecular inflammatory response in sjögren syndrome–associated dry eye patients under desiccating stress. Am J Ophthalmol. (2016) 161:133–41.e2. 10.1016/j.ajo.2015.09.039 26456254

[51] Sullivan DA Dana R Sullivan RM Krenzer KL Sahin A Arica B. Meibomian gland dysfunction in primary and secondary Sjogren syndrome. Ophthalmic Res. (2018) 59:193–205. 10.1159/000487487 29627826

[52] Sullivan DA Bélanger A Cermak JM Bérubé R Papas AS Sullivan RM. Are women with Sjögren's syndrome androgen-deficient? J Rheumatol. (2003) 30:2413–9. 14677186

[53] Valtysdóttir ST Wide L Hällgren R. Low serum dehydroepiandrosterone sulfate in women with primary Sjögren's syndrome as an isolated sign of impaired HPA axis function. J Rheumatol. (2001) 28:1259–65. 11409117

[54] Imperato-McGinley J Gautier T Cai L Yee B Epstein J Pochi P. The androgen control of sebum production. Studies of subjects with dihydrotestosterone deficiency and complete androgen insensitivity. J Clin Endocrinol Metab. (1993) 76:524–8. 10.1210/jcem.76.2.8381804 8381804

[55] Thody AJ Shuster S. Control and function of sebaceous glands. Physiol Rev. (1989) 69:383–416. 10.1152/physrev.1989.69.2.383 2648418

[56] Sullivan DA Sullivan BD Ullman MD Rocha EM Krenzer KL Cermak JM. Androgen influence on the meibomian gland. Invest Ophthalmol Vis Sci. (2000) 41:3732–42. 11053270

[57] Rocha EM Wickham LA da Silveira LA Krenzer KL Yu F-S Toda I. Identification of androgen receptor protein and 5α-reductase mRNA in human ocular tissues. Br J Ophthalmol. (2000) 84:76–84. 10.1136/bjo.84.1.76 10611104 PMC1723240

[58] Miyake K Ciletti N Liao S Rosenfield RL. Androgen receptor expression in the preputial gland and its sebocytes. J Invest Dermatol. (1994) 103:721–5. 10.1111/1523-1747.ep12398601 7963662

[59] Wickham LA Gao J Toda I Rocha EM Ono M Sullivan DA. Identification of androgen, estrogen and progesterone receptor mRNAs in the eye. Acta Ophthalmol Scand. (2000) 78:146–53. 10.1034/j.1600-0420.2000.078002146.x 10794246

[60] Sullivan DA Jensen RV Suzuki T Richards SM. Do sex steroids exert sex-specific and/or opposite effects on gene expression in lacrimal and meibomian glands? Mol Vis. (2009) 15:1553. 19693291 PMC2728565

[61] Wang L-X Deng Y-P. Androgen and meibomian gland dysfunction: from basic molecular biology to clinical applications. Int J Ophthalmol. (2021) 14:915. 10.18240/ijo.2021.06.18 34150548 PMC8165631

[62] Schirra F Gatzioufas Z Scheidt J Seitz B. Testosterone reduces the expression of keratinization-promoting genes in murine Meibomian glands. Der Ophthalmologe. (2013) 110:230–8. 10.1007/s00347-012-2661-5 23224122

[63] Schiffman R Bradford R Bunnell B Lai F Bernstein P Whitcup S. A multi–center, double–masked, randomized, vehicle–controlled, parallel group study to evaluate the safety and efficacy of testosterone ophthalmic solution in patients with meibomian gland dysfunction. Invest Ophthalmol Vis Sci. (2006) 47:5608.

[64] Sato E Ariga H Sullivan D. Impact of androgen therapy in Sjögren's syndrome: hormonal influence on lymphocyte populations and Ia expression in lacrimal glands of MRL/Mp-lpr/lpr mice. Invest Ophthalmol Vis Sci. (1992) 33:2537–45. 1634351

[65] Sato EA Matsumoto Y Dogru M Kaido M Wakamatsu T Ibrahim OM. Lacrimal gland in Sjögren's syndrome. Ophthalmology. (2010) 117:1055.e3. 10.1016/j.ophtha.2009.11.034 20438979

[66] Sullivan DA Edwards JA. Androgen stimulation of lacrimal gland function in mouse models of Sjögren's syndrome. J Steroid Biochem Mol Biol. (1997) 60:237–45. 10.1016/S0960-0760(96)00190-2 9191982

[67] Sullivan DA Rocha EM Aragona P Clayton JA Ding J Golebiowski B. TFOS DEWS II sex, gender, and hormones report. Ocul Surf. (2017) 15:284–333. 10.1016/j.jtos.2017.04.001 28736336

[68] Porola P Virkki L Przybyla BD Laine M Patterson TA Pihakari A. Androgen deficiency and defective intracrine processing of dehydroepiandrosterone in salivary glands in Sjögren's syndrome. J Rheumatol. (2008) 35:2229–35. 10.3899/jrheum.080220 18843777

[69] Guay A Munarriz R Jacobson J Talakoub L Traish A Quirk F. Serum androgen levels in healthy premenopausal women with and without sexual dysfunction: part A. Serum androgen levels in women aged 20–49 years with no complaints of sexual dysfunction. Int J Impot Res. (2004) 16:112–20. 10.1038/sj.ijir.3901178 14999217

[70] Park J Baek S. Dry eye syndrome in thyroid eye disease patients: the role of increased incomplete blinking and Meibomian gland loss. Acta ophthalmol. (2019) 97:e800–6. 10.1111/aos.14000 30593716

[71] Fenga C Aragona P Cacciola A Spinella R Di Nola C Ferreri F. Meibomian gland dysfunction and ocular discomfort in video display terminal workers. Eye. (2008) 22:91–5. 10.1038/sj.eye.6703025 17962818

[72] Lane SS DuBiner HB Epstein RJ Ernest PH Greiner JV Hardten DR. A new system, the LipiFlow, for the treatment of meibomian gland dysfunction. Cornea. (2012) 31:396–404. 10.1097/ICO.0b013e318239aaea 22222996

[73] Finis D Hayajneh J König C Borrelli M Schrader S Geerling G. Evaluation of an automated thermodynamic treatment (LipiFlow^®^) system for meibomian gland dysfunction: a prospective, randomized, observer-masked trial. Ocul Surf. (2014) 12:146–54. 10.1016/j.jtos.2013.12.001 24725326

[74] Godin MR Stinnett SS Gupta PK. Outcomes of thermal pulsation treatment for dry eye syndrome in patients with sjogren disease. Cornea. (2018) 37:1155–8. 10.1097/ICO.0000000000001621 29708939

[75] Epitropoulos AT Goslin K Bedi R Blackie CA. Meibomian gland dysfunction patients with novel Sjögren's syndrome biomarkers benefit significantly from a single vectored thermal pulsation procedure: a retrospective analysis. Clin Ophthalmol. (2017) 701–6. 10.2147/OPTH.S119926 28458508 PMC5402721

[76] Shen L Malyavantham K Suresh L Kapsogeorgou EK Tzioufas AG Sun Y. Early detection of sjögren's syndrome; sensitivity and specificity of the Sjö^®^ diagnostic test. Invest Ophthalmol Vis Sci. (2016) 57:5681.27784073

[77] Shen L Suresh L Sun Y Shi G. FRI0338 evaluation of the sensitivity and specificity of the sjÖ^®^ diagnostic test. Ann Rheum Dis. (2016) 75:557. 10.1136/annrheumdis-2016-eular.4216

[78] Di Marino M Conigliaro P Aiello F Valeri C Giannini C Mancino R. Combined low-level light therapy and intense pulsed light therapy for the treatment of dry eye in patients with Sjögren's syndrome. J Ophthalmol. (2021) 2021:2023246. 10.1155/2021/2023246 34221491 PMC8213457

[79] Huo Y Huang X Lin L Yang S Qin Z Yirui Z. The effect of intense pulsed light combined with topical 005% cyclosporin a eyedrops in the treatment of Sjögren's syndrome related dry eye. Expert Rev Clin Immunol. (2024) 20:1261–7. 10.1080/1744666X.2024.2358157 38785065

[80] Moon JY Yoon HJ Yoon K-C. Efficacy of intense pulsed light treatment in patients with Sjögren's syndrome associated with meibomian gland dysfunction. J Korean Ophthalmol Soc. (2021) 62:1581–91. 10.3341/jkos.2021.62.12.1581

[81] Huo Y Wan Q Hou X Zhang Z Zhao J Wu Z. Therapeutic effect of intense pulsed light in patients with Sjögren's syndrome related dry eye. J Clin Med. (2022) 11:1377. 10.3390/jcm11051377 35268468 PMC8911075

[82] Kim A Chong BF. Photosensitivity in cutaneous lupus erythematosus. Photodermatol Photoimmunol Photomed. (2013) 29:4–11. 10.1111/phpp.12018 23281691 PMC3539182

[83] Creadore A Watchmaker J Maymone MB Pappas L Vashi NA Lam C. Cosmetic treatment in patients with autoimmune connective tissue diseases: best practices for patients with lupus erythematosus. J Am Acad Dermatol. (2020) 83:343–63. 10.1016/j.jaad.2020.03.123 32360722

[84] Baxter SA Laibson PR. Punctal plugs in the management of dry eyes. Ocul Surf. (2004) 2:255–65. 10.1016/S1542-0124(12)70113-1 17216100

[85] Zang S Cui Y Cui Y Fei W. Meibomian gland dropout in Sjogren's syndrome and non-Sjogren's dry eye patients. Eye. (2018) 32:1681–7. 10.1038/s41433-018-0149-5 29934634 PMC6224582

[86] Noh SR Chung JL Lee JM Seo KY Koh K. Meibomian gland atrophy with duration of Sjogren's syndrome in adult females. Int Ophthalmol. (2022) 42:191–200. 10.1007/s10792-021-02013-7 34409540

